# Sparganosis, Henan Province, Central China

**DOI:** 10.3201/eid1701.101095

**Published:** 2011-01

**Authors:** Jing Cui, Xi Meng Lin, Hong Wei Zhang, Bian Li Xu, Zhong Quan Wang

**Affiliations:** Author affiliations: Zhengzhou University, Zhengzhou, People’s Republic of China (J. Cui, Z.Q. Wang);; Center for Disease Control of Henan Province, Zhengzhou (X.M. Lin, H.W. Zhang, B.L. Xu)

**Keywords:** Sparganosis, tadpoles, frogs, Rana spp., Spirometra, China, letter

**To the Editor:** Sparganosis is a parasitic zoonosis caused by invasion of the spargana, the plerocercoid larvae of various diphyllobothroid tapeworms belonging to the genus *Spirometra* (*1*). Although human sparganosis is cosmopolitan, it is most frequently found in eastern and southeastern Asia (*2*). During 1927–2009 in the People’s Republic of China, >1,000 cases in humans in 27 provinces were reported; most cases were in southern China, where human infections were mainly acquired by eating raw or insufficiently cooked meat of frogs and snakes or by placing frog or snake flesh on open wounds for treatment of skin ulcers or on eyes to treat inflammation (*3**,**4*).

Sparganosis is rarely seen in central and northern China. Before 2006, only 3 imported cases from southern China had been reported in Henan Province in central China (*5*). However, since 2006 in Henan Province, 20 autochthonous cases caused by ingestion of live tadpoles have emerged. To assess the risk for human infection with sparganosis in this province and to strengthen public safety awareness, we investigated spargana infection in the animal hosts of *Spirometra* tapeworms.

During July 2007–July 2010, wild frogs and frog tadpoles were collected from the cities of Shangqiu, Zhoukou, and Luohe in Henan Province. Necropsies identified plerocercoids in 11.93% (163/1,366) of tadpoles and in 26.58% (172/647) of frogs. By frog species, plerocercoids were found in 31.09% (111/357) of *Rana limmocharis* and 26.29% (61/232) of *R. nigromaculata* frogs, each significantly (p<0.05) more numerous in these species than in *R. temporaria* frogs (0/58). In addition, 177 wild frogs sold at markets in Luohe were also examined; plerocercoids were detected in 30.39% (31/102) of *R. limmocharis* and 28% (21/75) of *R. nigromaculata* frogs. Thus, in Henan Province, *R. limmocharis* and *R. nigromaculata* frogs are the main intermediate hosts of *Spirometra* tapeworms.

No pathologic changes associated with the tapeworms were found in dissected tadpoles and frogs. We found 250 plerocercoids in tadpoles and 1,387 in frogs. Tadpoles contained 1–14 (mean 1.53) and frogs 1–87 (mean 6.85) tapeworms. In frogs, most plerocercoids were located in the muscles of hind legs and back; some were in the muscles of the abdominal wall and forelegs. Plerocercoids dissected from tadpole and frog tissues were wrinkled, whitish, and ribbon-shaped and continuously moved while in normal saline. Plerocercoids from tadpoles were 1–8 mm long and 0.2–0.5 mm wide; those from frogs were 1–13 cm long and 1–2.5 mm wide.

Cyclops were collected from ponds and ditches by using a 425-µm mesh (no. 40) sieve and species were identified by microscopic appearance as *Mesocyclops leuckarti* (*6**,**7*). Procercoids were microscopically found in the hemocele of 3.53% (3/85) of cyclops; 3–5 worms per cyclop were found.

Fecal examination of dogs and cats found *Spirometra mansoni* tapeworm eggs in 19.35% (6/31) of dogs and 33.33% (1/3) cats. In addition, a 3-month-old specific pathogen–free cat was orally inoculated with 33 plerocercoids from tadpoles, fecal samples were microscopically examined by sedimentation during 10–25 days postinfection (dpi), and the cat was euthanized and examined for adult worms. *S. mansoni* tapeworm eggs were found in the feces during 12–25 dpi, and 17 adult worms, 26–45 cm long, were recovered from the small intestines at 25 dpi; however, no plerocercoids were seen in the tissues. The adult worms were morphologically identified as *S. mansoni* according to the following features: scolex with 2 longitudinal grooves, mature and gravid proglottids with conspicuous uterus at the center of segments, spiral-shaped uterus (unlike the rosette-shaped uterus of *Diphyllobothrium latum*), and cirrus and vaginal pore with separate openings (*8**,**9*).

Although in recent years in Henan Province wild frogs have been sold clandestinely at markets, persons in this province do not routinely eat raw frog meat or use raw meat as poultices. However, some villagers in Henan do believe that eating live tadpoles has a medicinal role for skin diseases and, thus, they contract sparganosis. Accordingly, the route of plerocercoid infection for humans in Henan Province differs from that in southern China. Because this disease is rare in central and northern China, sparganosis is often neglected and misdiagnosed. Our survey showed that in Henan Province, 11.93% of tadpoles are infected with plerocercoids and 3.53% of cyclops are infected with procercoids. Therefore, eating live tadpoles poses a high risk for plerocercoid infection and must be discouraged. In addition, drinking raw water containing cyclops also poses a slight risk for sparganosis.
